# Effects of sprint interval and continuous endurance training on serum levels of inflammatory biomarkers

**DOI:** 10.1186/2251-6581-12-22

**Published:** 2013-05-31

**Authors:** Fariborz Hovanloo, Tahereh Arefirad, Sajad Ahmadizad

**Affiliations:** 1 Faculty of Physical Education and Sport Sciences, Shahid Beheshti University, Tehran, 1983963113, Iran; 2 Faculty of Physical Education and Sport Sciences, Shahid Beheshti University, Evin Tehran, 02129902941, Iran

**Keywords:** Inflammatory biomarkers, Sprint interval training, Continuous endurance training

## Abstract

Chronic and inflammatory diseases are major causes of mortality. Although the anti-inflammatory effects of exercise have been confirmed, but the effect of different types of exercise on inflammatory markers is different. The aim of this study is comparing the effects of two types of sprint interval (SIT) and continuous endurance (CET) training on inflammatory markers. Sixteen students who had recreational activities participated in this study and were randomly assigned to one of the two protocols. The SIT protocol consisted of four to six 30-s “all-out” Wingate tests separated by 4 minutes of recovery and The CET protocol included 90–120 minutes of cycling at 65% Vo2max. The two protocols were performed 3 days per week and for two weeks. In each group, two blood samples were collected before and 2 days (24 and 48 hrs) after the training. Results showed that there was no significant difference between the two training protocols on all measured parameters (p>0.05). The results of present study showed that the SIT and CET have identical effects on inflammatory markers.

## Introduction

Chronic and inflammatory diseases are major causes of mortality [1]. On the other hand, anti-inflammatory effects of exercise have been confirmed [2-4] and its effect has been shown on pro and anti-inflammatory cytokines [5,6], and thus, the protective effect of exercise against mortality is quite plausible. One study has demonstrated a strong and consistent inverse relationship between physical fitness and leukocyte count and markers of inflammation such as serum IL-6 and CRP, whereas serum level IL-10 is positively correlated with fitness [2]. Fischer et al. (2004) have shown that mRNA expression content of exercise-induced IL-6 is markedly reduced in human skeletal muscle after 10 wk endurance training [7], but Robson-Ansley et al. (2007) have shown that plasma level IL-6 content is significantly elevated following intensified training [8]. Although current recommendations for physical fitness involve performing aerobic and resistance exercise with moderate to vigorous intensity for several hours per week, people generally fail to follow such regimes due to lack of time [9]. Nowadays, experts have focused on interval training program with high intensity and low volume [10]. Previous studies have shown that this training induces fat oxidation during training in women [11], improves insulin action in young sedentary men[9] and improves muscle oxidative capacity [10,12,13] and it is superior to moderate intensity exercise in rats[14]. Rakobowchuk et al. (2008) have also confirmed that 6-week sprint interval training with low volumeis a time-efficient strategy to elicit improvements in peripheral vascular structure and function that are comparable to endurance training [15]. Some researchershave compared the effects of sprint interval training (SIT) with continuous endurance training (CET) on inflammatory markers, but the results have been controversial. The aim of the current study is to examine whether there is any significant difference between the effect of sprint interval and continuous endurance training on inflammatory markers.

## Methods

### Subjects

In this randomized trial, 16 students (8 men, 8 women) who had regular recreational activities participated. All subjects took some form of recreational exercise two to three times per week (jogging, cycling, etc.). Subjects' completed two questionnaires before starting the training program: one about baseline information and the other about the type and intensity of their physical activity. Subjects were randomly assigned to either the SIT or the CET training program. Following routine medical screening, the purpose of the study and associated risks were explained for all subjects and written informed consent was obtained from them. The study was approved by the Research Ethics Committee of Shahid Beheshti University.

### Pre-experimental procedures

Prior to baseline measurements, subjects were make several familiarization visited the laboratory in order to get informed of the testing procedures and training devices. During one of these visits, subjects performed an incremental test to exhaustion on an electronically braked cycle ergometer using an online gas collection system to determine Vo_2max_. Following a 5-minute warm-up, the test began at 50W with the workload increasing by 25Wevery 2 minutes until volitional exhaustion. The value recorded for Vo_2max_corresponded to the highest value achieved over a 30-second collection period [13]. All exercise tests were performed at least 1week prior to baseline testing. During the test, heart rate was measured continuously using a heart rate monitor. Vo_2max_ was confirmed using established physiological criteria defined by the British Association of Sport and Exercise Science (BASES), and included a respiratory exchange ratio (RER) above 1.15, oxygen uptake reaching a plateau with increasing work rate, and a heart rate close to age predicated maximal values [16].

### Wingate test

Subjects in the SIT group completed a 30second maximal effort on an electronically braked cycle ergometer at a resistance equivalent to 7.5% of their body mass.

### Training protocol

CET consisted of continuous cycling on an ergometer, 3 days per week for2 weeks, at a power output corresponding to 65% Vo_2max_. Subjects did exercise training in the first two sessions for90 minutes, in the second and third sessions for 105 minutes, and in the last two sessions for 120 minutes. The numbers of Wingate tests performed during each training session were increased from four in sessions 1 and 2 to five in sessions 3 and 4, and finally to six in sessions 5 and 6. For all training sessions, the recovery interval between Wingate tests was fixed at 4 minutes during which, subjects rested[10] (Table 1).

**Table 1:** Continuous endurance training (CET) and sprint interval training protocol

**Group**	**Sessions**	**1 1**	**22**	**3 3**	**4 4**	**5 5**	**6**
CET	Total time (min)	90	90	105	105	120	120
SIT	Wingate tests	4	4	5	5	6	6
	Exercise time (min)	2	2	2.30	2.30	3	3
	Recovery (n)	3	3	4	4	5	5

### Blood sampling and analyses

Venous blood samples were obtained at 8–10 am in the Obesity Research Center, Research Institute for Endocrine Sciences of Shahid Beheshti University of Medical Sciences. Subjects had slept and had fasted for 8–9 hours preceding blood sampling, eat food before 2 blood sampling as same as possible, and were asked not to do heavy exercise, take medications, or get exposed to sensitizers^a^ within 24 hours preceding blood sampling. To be included in the study, subjects ought to have had no infection within one week before sampling. Blood samples were taken after 15 minutes of rest in sitting position. The first samples were taken 24 h before the first session and the second samples were taken 48 h after the last session (after 2 weeks). Blood samples (10 ml) were obtained from antecubital vein. Two ml of the blood sample was poured into EDTA tubes for WBC measurement and the rest was centrifuged at 4C for 5 minutes at 3000 g. After centrifugation, serum was stored at –70C for subsequent analyses.CRP, IL-10, IL-6were measured by ELISA kitand insulin resistance was calculated as HOMA-IR.

### Statistical analyses

The distribution of variables was assessed by kolmogorov-Smirnov test. To compare groups, we calculated the difference (after- before) for each group and tested it by T test if the distribution was normal and by Mann- Whitney U test if the distribution was not normal. The before and after mean in each group was tested by Paired T test if the distribution was normal and by Wilcoxon test if the distribution was not normal. P-values less than 0.05 were considered statistically significant. Analysis was done with SPSS, PC program, version 16. (SPSS Inc., IIIinois Chicago, USA).

## Results

All variables with the exception of CRP had normal distribution. The baseline characteristics of subject were not statistically different between two groups (Table 2). Results showed that the type and duration of training had no significant effect on IL-6, IL-10, CRP, WBC and insulin resistance index (P>0.05). The level of IL-6 was different between the two training groups. Although mean level of IL-6 increased after CET and decreased after SIT, it was not statistically significant (P>0.05). Results showed that serum levels of IL-10 and CRP decreased after both training programs but the decrease was not statistically significant (P>0.05). WBC also decreased after both training programs, but the decrease was significant only after CET (P:0.03). In addition,the decrease in insulin resistance index was close to significance after SIT (P:0.053) but it showed no change in CET (P: 0.94) (Tables 3, 4).

**Table 2:** Baseline characteristics of subjects in two group

**Variable**	**Group**		**P-value**
	**CET**	**SIT**	
Age(year)	25(1.69)	22(2.16)	0.07
Weight (kg)	65.38(1.14)	65.91(7.88)	0.96
Height(Cm)	1.70(0.09)	1.71(0.09)	0.70
BMI(kg/m2)	22.44(2.02)	22.37(2.50)	0.95
VO2max(ml/kg/min)	34.17(5.52)	33.75(6.08)	0.88

The data are expressed as mean (SD).

**Table 3:** Inflammatory biomarkers mean in before and after of two group

**Type of training**	**CET (n=8)**			**SIT (n=8)**		
	**Before**	**After**	**P-value**	**Before**	**After**	**P-value**
IL-6_(pg/ml)_	2.88(0.21)	2.91(0.45)	0.87	3.00(0.41)	2.78(0.26)	0.33
IL-10_(pg/ml)_	7.95(0.96)	7.85(1.15)	0.87	7.60(1.64)	7.21(1.46)	0.64
CRP_(ng/ml)_	461.40(294)	333(361.34)	0.20	373.5(144.66)	341.66(325.06)	0.09
WBC_(×10_^3^_/μl)_	5.97(0.99)	5.35(0.89)	0.03	6.08(1.12)	6.02(1.55)	0.9
HOMA-IR	1.17(0.57)	1.17(0.70)	0.94	1.78(0.58)	1.24(0.32)	0.053

The data are expressed as mean (SD).

**Table 4:** Inflammatory biomarkers mean in before and after of two groups

**Type of training**	**CET (n=8)**	**SIT (n=8)**	**P-value**
	**Difference (After – Before)**	**Difference (After – Before)**	
IL-6_(pg/ml)_	0.03 (0.46)	−0.21(0.54)	0.38
IL-10_(pg/ml)_	−0.10(1.80)	−0.38(2.11)	0.78
CRP_(ng/ml)_	−160(220)	−31.83(347.70)	0.75
WBC_(×10_^3^_/μl)_	−0.62(0.69)	−0.06(1.50)	0.35
HOMA-IR	0.01(0.22)	−0.53(0.65)	0.06

The data are expressed as mean (SD).

## Discussion

The major finding of the present study was that neither CET nor SIT has any significant effect on serum levels of I-6, IL-10, CRP, WBC, and insulin resistance index. To the best of our knowledge, this is the first study on the effects of two types of interval and continuous training with identical format (cycling), frequency (3 days in the week), and duration (2 weeks) but different volume. Previous studies have investigated effects of either interval or continuous training on these markers [7,17-22] but none has studied the effects of both trainings simultaneously.

Our results are consistent with results reported by Rakobowchuk et al. (2008) and Gibala et al. (2006) that showed no difference between these two training types[10,15]. However, Rakobowchuk had studied untrained subjects with 4.5 min recovery periods. Tejona et al. (2008) had reported that interval training is more efficient than continuous training in improving metabolic parameters in patients with metabolic syndrome [23]. However; the results may have been biased by differences between subjects, protocol and time (16 weeks). Most studies have showed improvements in these cytokines by 6 to 24 weeks of training [7,19-21,24]; therefore, the time required for these changes should be more than 2 weeks. Muscles are sources of IL-6 in during exercise [25]. In the present study; we have not taken biopsies from muscles. Previous results have shown that IL-6 response is different between the 2 types of training that may be due to gender as a determinant [26,27]. Another justification is that myofibrils have differences in expression of cytokines [24] and differences between 2 types of training may be due to difference in involvement of slow and fast fibrils. Similarly, Gibala et al. (2006) and Burgomasteret al (2005) reported that SIT can increase glycogen content and muscle oxidative capacity [10,13], for this reason mussels didn’t rely on to IL-6 and its effects about provided substrate from liver and fat tissue [28] and decreased levels IL-6 in this research but CET increased IL-6 that its reason can decrease glycogen content. Low glycogen content is most important factor for this increase probably [29-31] that this situation mostly body rely on fatty acid and IL-6 that increased lipolysis. In the other hand, long activity induced injured mussels and there is a inflammatory response and produced this cytokine [8]. CRP decreased in both types of training, because IL-6 can induce CRP [32], therefore the decrease in CRP is expected in SIT but not in CET. As Oberbach et al. (2006) reported, we can conclude that CRP is not induces only by IL-6 and other factors can also induce production of CRP [33]. In the present study, IL-10 decreased after both types of training, this cytokine controls inflammation by suppressing the production of pro-inflammatory cytokines [34] but other changes related to regular training such as decreased weight or percent body fat can justify the anti-inflammatory effects of training that has not been investigated in this study [33]. Moreover, IL-10 is produced by T cells, B cells, monocytes and macrophages as well [35], therefore decreased WBC after both types of training can induce decrease in IL-10. The decrease in WBC in CET was more prominent than SIT which may be due to Fitness [36] and increased plasma volumes after endurance training [37] can explain decreased WBC. Finally, insulin resistance index decreased in SIT but did not change in CET. Increased oxidative capacity of muscles, less dependence of body on IL-6, and increased sensitivity to IL-6, lead to decreased requirement of the body to glucose in blood. However, increased IL-6 after CET showed that adoptions after training have not been sufficient for the required activity within muscles and substrate should have been provided to cells from outside, which explains why no decrease in insulin resistance index was observed. Effects of CET remained after the last session for a short time period because half-life of 4 GLUT is short [38]. Therefore, 48h blood sampling has been too late for this measurement. The main limitation of this study is low sample size which decrease power of current study. The decrease in IL-6 and CRP serum levels after SIT requires studies with longer follow-up (more than 6 sessions) in order to improve health status of people by means of shorter but more efficient training.

## Conclusion

Our results shows that SIT with high intensity and low volume, and CET with moderate intensity and high volume have identical effects on the body. There were no significant improvements in inflammatory biomarkers, but the decrease in insulin resistance index was marginally significant.

## Endnote

^a^Any substance to which subjects were sensitive and had mentioned it in the questionnaire.

## Competing interests

The authors declared that they have no competing interests.

## Authors’ contribution

FH participated in study designed and drafted the manuscript. TA participated in study designed performing laboratory tests, data acquisition and drafted the manuscript. SA participated in study designed and statistical analysis. All authors read and approved the final manuscript.

## References

[B1] Anne M Petersen W Pedersen BK The anti-inflammatory effect of exercise J Appl Physiol 2005 98 1154 1162 10.1152/japplphysiol.00164.2004 15772055

[B2] Markovitch D Tyrrell R Thompson D Acute moderate-intensity exercise in middle-aged men has neither an anti-nor proinflammatory effect J Appl Physiol 2008 105 1 260 265 10.1152/japplphysiol.00096.2008 18467550 PMC2494829

[B3] Kasapis C Thompson P The Effects of Physical Activity on Serum C-Reactive Protein and Inflammatory Markers: A Systematic Review J Am Coll Cardiol 2005 45 10 1563 1569 10.1016/j.jacc.2004.12.077 15893167

[B4] Meilhac O Ramachandran S Chiang K Santanam N Parthasarathy S Role of arterial wall antioxdant defense in beneficial effects of exercise on atherosclerosis in mice Arterioscler ThrombVasc Biol 2001 21 1681 1688 10.1161/hq1001.097106 11597945

[B5] Haider D Pleiner J Francesconi M Wiesinger G Muller M Wolzt M Exercise training lowers plasma visfatin concentrations in patients with type 1 diabetes J Clin Endocrinol Metab 2006 91 11 4702 10.1210/jc.2006-1013 16895956

[B6] Frydelund-Larsen L Akerstrom T Nielsen S Keller P Keller C Pedersen BK Visfatin mRNA expression in human subcutaneous adipose tissue is regulated by exercise Am J Physiol Endocrinol Metab 2007 292 1 24 31 10.1152/ajpendo.00113.2006 16868228

[B7] Fischer CP Plomgaard P Hansen AK Pilegaard H Saltin B Pedersen BK Endurance training reduces the contraction-induced interleukin-6 mRNA expression in human skeletal muscle Am J Physiol Endocrinol Metab 2004 287 6 E1189 E1194 10.1152/ajpendo.00206.2004 15304377

[B8] Robson-Ansley PJ Blannin A Gleeson M Elevated plasma interleukin-6 levels in trained male triathletes following an acute period of intense interval training Eur J Appl Physiol 2007 99 4 353 360 10.1007/s00421-006-0354-y 17165057

[B9] Babraj JA Vollaard NB Keast C Guppy FM Cottrell G Timmons JA Extremely short duration high intensity interval training substantially improves insulin action in young healty males BMC Endocr Disord 2009 9 1 8 10.1186/1472-6823-9-1 19175906 PMC2640399

[B10] Gibala MJ Little JP Van Essen M Wilkin GP Burgomaster KA Safdar A Raha S Tarnopolsky MA Short-term sprint interval versus traditional endurance training: similar initial adaptations in human skeletal muscle and exercise performance J Physiol 2006 575 3 901 911 10.1113/jphysiol.2006.112094 16825308 PMC1995688

[B11] Talanian JL Galloway SD Heigenhauser GJ Bonen A Spriet LL Two weeks of high-intensity aerobic interval training increase the capacity for fat oxidation during exercise in women J Appl Physiol 2007 102 1439 1447 17170203 10.1152/japplphysiol.01098.2006

[B12] Burgomaster KA Howarth KR Phillips SM Rakobowchuk M Macdonald MJ McGee SL Similar metabolic adaptations during exercise after low volume sprint interval and traditional endurance training in humans J Physiol 2008 586 1 151 160 17991697 10.1113/jphysiol.2007.142109 PMC2375551

[B13] Burgomaster KA Hughes SC Heigenhauser GJ Bradwell SN Gibala MJ Six sessions of sprint interval training lincreases muscle oxidative potential and cycle endurance capacity in humans J Appl Physiol 1985 98 6 1985 1990 15705728 10.1152/japplphysiol.01095.2004

[B14] Haram PM Kemi OJ Lee SJ Bendheim MO Al-Share QY Waldum HL Aerobic interval training vs. continuous moderate exercise in the metabolic syndrome of rats artificially selected for low aerobic capacity Cardiovasc Res 2009 81 4 723 732 19047339 10.1093/cvr/cvn332 PMC2642601

[B15] Rakobowchuk M Tanguay S Burgomaster KA Howarth KR Gibala MJ MacDonald MJ Sprint interval and traditional endurance training induce similar improvements in peripheral arterial stiffness and flow-mediated dilation in healthy humans Am J Physiol Regul Integr Comp Physiol 2008 295 1 236 242 10.1152/ajpregu.00069.2008 PMC2494806 18434437

[B16] Bassami M Ahmadizad S Doran D MacLaren DP Effects of exercise intensity and duration on fat metabolism in trained and untrained older males Eur J Appl Physiol 2007 101 4 525 532 10.1007/s00421-007-0523-7 17724610

[B17] Hatunic M Finucane F Burns N Gasparro D Nolan JJ Vascular inflammatory markers in early-onset obese and type 2 diabetes subjects before and after three months' aerobic exercise training Diab Vasc Dis Res 2007 4 3 231 234 17907114 10.3132/dvdr.2007.045

[B18] Kohut ML McCann DA Russell DW Konopka DN Cunnick JE Franke WD Aerobic exercise, but not flexibility/resistance exercise, reduces serum IL-18 CRP, and IL-6 independent of beta-blockers, BMI, and psychosocial factors in older adults Brain Behav Immun 2006 20 3 201 209 10.1016/j.bbi.2005.12.002 16504463

[B19] Thompson D Markovitch D Betts JA Mazzatti D Turner J Tyrrell RM Time course of changes in inflammatory markers during a 6-mo exercise intervention in sedentary middle-aged men: a randomized-controlled trial J Appl Physiol 2007 108 4 769 779 20368384 10.1152/japplphysiol.00822.2009

[B20] Bruun JM Helge JW Richelsen B Stallknecht B Diet and exercise reduce low-grade inflammation and macrophage infiltration in adipose tissue but not in skeletal muscle in severely obese subjects Am J Physiol Endocrinol Metab 2006 290 5 961 967 10.1152/ajpendo.00506.2005 16352667

[B21] Croft L Bartlett JD MacLaren DP Reilly T Evans L Mattey DL High-intensity interval training attenuates the exercise-induced increase in plasma IL-6 in response to acute exercise Appl Physiol Nutr Metab 2009 34 6 1098 1107 10.1139/H09-117 20029520

[B22] Christiansen T Paulsen SK Bruun JM Pedersen SB Richelsen B Exercise training versus diet-induced weight-loss on metabolic risk factors and inflammatory markers in obese subjects: a 12-week randomized intervention study Am J Physiol Endocrinol Metab 2010 298 4 E824 E831 10.1152/ajpendo.00574.2009 20086201

[B23] Tjonna AE Lee SJ Rognmo O Stolen TO Bye A Haram PM Aerobic interval training versus continuous moderate exercise as a treatment for the metabolic syndrome: a pilot study Circulation 2008 118 4 346 354 10.1161/CIRCULATIONAHA.108.772822 18606913 PMC2777731

[B24] Lira FS Koyama CH Yamashita AS Rosa JC Zanchi NE Batista ML Chronic exercise decreases cytokine production in healthy rat skeletal muscle Cell Biochem Funct 2009 27 7 458 461 10.1002/cbf.1594 19681095

[B25] Febbraio MA Pedersen BK Muscle-derived interleukin-6: mechanisms for activation and possible biological roles FASEB J 2002 16 1335 1347 10.1096/fj.01-0876rev 12205025

[B26] Moyna NM Acker GR Fulton JR Weber K Goss FL Robertson RJ Tollerud DJ Rabin BS Lymphocyte function and cytokine production during incremental exercise in active and sedentary males and females Int J Sports Med 1996 17 8 585 591 10.1055/s-2007-972899 8973979

[B27] Edwards KM Burns VE Ring C Carroll D **Individual differences in the interleukin-6 response to maximal and submaximal exercise ta**sks J Sports Sci 2006 24 8 855 862 10.1080/02640410500245645 16815780

[B28] Keller C Steensberg A Hansen AK Fischer CP Plomgaard P Pedersen BK Effect of exercise, training, and glycogen availability on IL-6 receptor expression in human skeletal muscle J Appl Physiol 2005 99 6 2075 2079 10.1152/japplphysiol.00590.2005 16099893

[B29] Pedersen BK Steensberg A Fischer C Keller C Keller P Plomgaard P Wolsk-Petersen E Febbraio M **The metabolic role of IL-6 produced during exercise: is IL-6 an exercise factor**? Proc Nutr Soc 2004 63 2 263 267 10.1079/PNS2004338 15294041

[B30] Ronsen O Lea T Bahr R Pedersen BK Enhanced plasma IL-6 and IL-1ra responses to repeated vs. single bouts of prolonged cycling in elite athletes J Appl Physiol 2012 92 6 2547 2553 12015372 10.1152/japplphysiol.01263.2001

[B31] Holmes AG Watt MJ Febbraio MA Suppressing lipolysis increases interleukin-6 at rest and during prolonged moderate-intensity exercise in humans J Appl Physiol 2004 97 2 689 696 10.1152/japplphysiol.00195.2004 15075299

[B32] Puglisi MJ Fernandez ML Modulation of C-reactive protein, tumor necrosis factor-alpha, and adiponectin by diet, exercise, and weight loss J Nutr 2008 138 12 2293 2296 10.3945/jn.108.097188 19022947

[B33] Oberbach A Tonjes A Kloting N Fasshauer M Kratzsch J Busse MW Paschke R Stumvoll M Bluher M Effect of a 4 week physical training program on plasma concentration of inflammatory markers in patients with abnormal glucose tolerance Eur J Endocrinol 2006 154 577 585 10.1530/eje.1.02127 16556721

[B34] Rowsey PJ Metzger BL Carlson J Gordon CJ Long-term exercise training selectively alters serum cytokines involved in fever Biol Res Nurs 2009 10(4 374 380 19190031 10.1177/1099800408329409

[B35] Esposito K Pontillo A Giugliano F Giugliano G Marfella R Nicoletti G Giugliano D Association of low interleukin-10 levels with the metabolic syndrome in obese women J Clin Endocrinol Metab 2003 88 3 1055 1058 10.1210/jc.2002-021437 12629085

[B36] Tanimura Y Kon M Shimizu K Kimura F Kono I Ajisaka R Effect of 6-day intense Kendo training on lymphocyte counts and its expression of CD95 Eur J Appl Physiol 2009 107 2 227 233 10.1007/s00421-009-1119-1 19568765

[B37] Convertino VA Blood volume: its adaptation to endurance training Med Sci Sports Exerc 1991 23 12 1338 1348 1798375

[B38] Devries MC Hamadeh MJ Glover AW Raha S Samjoo IA Tarnopolsky MA Endurance training without weight loss lowers systemic, but not muscle, oxidative stress with no effect on inflammation in lean and obese women Free Radic Biol Med 2008 45 4 503 511 10.1016/j.freeradbiomed.2008.04.039 18502211

